# A Study on the Drug Concentration in Fellow Eyes After Unilateral Intravitreal Injection of Conbercept Into New Zealand Rabbit Eyes

**DOI:** 10.3389/fphar.2021.783057

**Published:** 2021-12-01

**Authors:** Yu Di, Haiyan Xu, Junjie Ye, Zijian Guo

**Affiliations:** ^1^ Department of Ophthalmology, Peking Union Medical College Hospital, Chinese Academy of Medical Sciences, Beijing, China; ^2^ Department of Clinical Laboratory, Peking Union Medical College Hospital, Chinese Academy of Medical Sciences, Beijing, China

**Keywords:** conbercept, non-injected eyes, vitreous, aqueous humor, serum, drug concentration

## Abstract

Intravitreal injections of anti-vascular endothelial growth factor (VEGF) have become increasingly popular in the treatment of ocular diseases. However, few studies have determined the efficiency of unilateral intravitreal anti-VEGF injection in the fellow eye. Herein, we performed a study to investigate the drug concentration in fellow eyes and venous serum after unilateral intravitreal injection of conbercept into rabbit eyes. This is an experimental animal study. Thirty male New Zealand rabbits (60 eyes) were used. One eye of each rabbit was intravitreally injected with 0.5 mg of conbercept. Both eyes from six rabbits were enucleated on days 1, 3, 7, 14, and 30. Conbercept concentrations were measured in the serum, aqueous humor, and vitreous humor. We found conbercept was detected in the fellow eyes and serum of rabbits. Conbercept concentrations in the vitreous humor of the fellow eyes increased from 74.11 ng/ml on day 1 to 246.69 ng/ml on day 3 and then declined to 69.11 ng/ml after 30 days. The concentration in the aqueous humor peaked on day 1 with a concentration of 244.82 ng/ml and declined to 40.13 ng/ml after 30 days. The maximum conbercept concentrations in the aqueous humor and vitreous humor of fellow eyes were similar, which were 0.2 and 1.3% of those of the injected eye, respectively. A peak concentration of 102.49 ng/ml was achieved in the venous serum 1 day after intravitreal injection of conbercept, which was 0.08 and 0.5% of those of the maximum conbercept concentrations in the vitreous humor and aqueous humor of the injected eye, respectively, and 41.5 and 41.8% of the maximum conbercept concentrations in the vitreous humor and aqueous humor of the non-injected eye, respectively. In conclusion, after intravitreal injection of 0.5 mg of conbercept into rabbit eyes, very small amounts of conbercept were detected in the fellow non-injected eyes and venous serum.

## Introduction

Intravitreal injections of anti-vascular endothelial growth factor (VEGF) have become increasingly popular in the treatment of ocular illness such as neovascular age-related degeneration (nAMD), diabetic macular edema (DME), and macular edema secondary to retinal vein occlusion in recent years. Its safety and efficiency have been shown by a large number of clinical trials (Pozarowska and Pozarowski, 2016). The novel anti-VEGF reagent conbercept (KH902; Chengdu Kanghong Biotech Co., Sichuan, China) is a fully humanized, soluble, VEGF receptor protein. It contains extracellular domain 2 of vascular endothelial growth factor receptor (VEGFR) 1 (Flt-1) and extracellular domains 3 and 4 of VEGFR2 (KDR-3 and 4) fused to the Fc portion of the human immunoglobulin G1 (IgG1). The most notable feature of conbercept is that it binds to VEGF-A, VEGF-B, and the placental growth factor with high affinity. Extracellular domain 4 of VEGFR2 can stabilize the receptor–ligand complex and lengthen the half-life of conbercept to more effectively reduce vitreous VEGF concentrations and inhibit neovascularization (Chen et al., 2013).

However, few studies have determined the efficiency of unilateral intravitreal anti-VEGF injection in fellow eyes (Avery et al., 2014; Huang et al., 2018). In patients with bilateral DME, we investigated the best corrected visual acuity (BCVA) and central retinal thickness (CRT) changed in untreated eyes after receiving the unilateral intravitreal conbercept injection. At the last visit, CRT decreased significantly in the untreated eyes and increased/unchanged BCVA accounted for a large proportion of the eyes (67%) after treatment (Di et al., 2020). Nevertheless, the anti-VEGF mechanism in untreated eyes is controversial at present. In a rabbit model, Bakri et al. (2007a, b) reported the pharmacokinetics of intravitreal bevacizumab and ranibizumab. In contrast to intravitreal ranibizumab, which was not detectable in the non-injected eyes during the follow-up, these authors found that a low concentration of bevacizumab enters systemic circulation and can be detected 4 weeks later in the aqueous humor of the non-injected eyes. In this study, intravitreal injection of conbercept was applied unilaterally to rabbit eyes, and the drug concentration in the venous serum, the aqueous humor, and the vitreous humor of the fellow eyes was detected to further analyze the mechanism of conbercept in fellow rabbit eyes.

## Materials and Methods

### Animals

Thirty male New Zealand rabbits weighing 2.5–3 kg were used. A single intravitreal injection of conbercept (0.5 mg/0.05 ml) was administered in the right eyes of all rabbits, and the left eyes received no intravitreal injection. All animal research conformed to the Laboratory Animal Ethics Committee of Peking Union Medical College Hospital (XHDW-2020–039), and the procedures adhered to the guidelines from the Association for Research in Vision and Ophthalmology for animal use in research.

### Surgical Procedures

The rabbits were anesthetized with 10 mg/kg of intramuscular injection of xylazine hydrochloride (2.0 ml, Huamu Animal Health Products Co. Ltd., Jilin, China) and 0.4% oxybuprocaine hydrochloride ophthalmic drops (5.0 ml, Santen Pharmaceutical Co., Ltd., Japan) topically on the eye. Povidone iodine (5%, 60 ml, Likang Disinfection Technology Co., Ltd., Shanghai, China) was placed on the conjunctiva of injected eyes. The vitreous puncture site, 2.5 mm behind the superior nasal limbus, was determined by the Castroviejo Caliper curved ophthalmic ruler. Then, a 30-gauge needle was used to inject intravitreally through the puncture site, and the tip was directed into the mid-vitreous about 6 mm deep. At last, 0.5 mg (0.05 ml) of conbercept was slowly administered. Ofloxacin eye ointment (3.5 g, Santen Pharmaceutical Co., Ltd., Japan) was topically applied once immediately after the procedure. Eyes were monitored weekly for signs of inflammation.

### Specimen Acquisition and Processing

Six rabbits were sacrificed by 100 mg/kg intravenous pentobarbital sodium (Sino Pharm Chemical Reagent Co. Ltd., China) on each of the following days: days 1, 3, 7, 14, and 30. A venous blood sample was taken from each rabbit. Both eyes were enucleated, and 0.5 ml of aqueous humor and 1.0 ml of vitreous humor were extracted into a syringe using 30-gauge and 18-gauge needles, respectively. The samples of aqueous humor and vitreous humor were then frozen at -80°C. The serum was collected by centrifugation (4,000 r/min) for 5 min after allowing the sample to clot at room temperature for 1 h. The serum was frozen at -80°C until testing. Before the immunoassay, all samples were diluted as needed.

### Conbercept Assay

The enzyme-linked immunosorbent assay (ELISA) method was used to measure conbercept concentrations. Human VEGF 100 protein (ACROBiosystems, Newark, DE, United States) was used for capturing, and horseradish peroxidase–conjugated AffiniPure rabbit anti-human IgG (Boster Biological Technology, Co., Ltd., Wuhan, China) was used for detection. The human VEGF 100 protein was diluted to a concentration of 1 μg/ml in PBS and then aliquoted into 96-well microplates (Greiner Bio-One GmbH, Kremsmünster, Austria) at a concentration of 100 μl/well. After incubating overnight at 4°C, the microplates were washed three times with PBST and blocked for 2 h at room temperature with 1% bovine serum albumin (BSA)-PBS. The plates were stored dry at 4°C after a final wash with PBS.

Samples were diluted in PBST and aliquoted onto VEGF microplates at 100 μl/well, and then, the microplates were shaken for 1 h at room temperature. For each individual assay, a standard curve was constructed using conbercept at known concentrations (0, 3, 10, 30, 100, 300, and 1,000 ng/ml). After the initial shaking of the microplates, they were washed five times with PBST (350 μL/well). Horseradish peroxidase–conjugated AffiniPure rabbit anti-human IgG was diluted 1:5,000 ∼ 1:10,000 in 0.2% BSA-PBS and then aliquoted onto microplates at 100 μl/well. Then, the microplates were shaken for 1 h at room temperature and washed five times with PBST (350 μL/well). The TMB substrate was aliquoted onto the microplates at 100 μl/well and then shaken for 10–20 min at room temperature and in the dark. The process was then stopped by adding phosphoric acid, and the absorption was measured at 450 nm with a reference wavelength of 620 nm. The concentration of conbercept was determined using the same standard curve’s four-parameter fit.

### Pharmacokinetic Methods

Conbercept aqueous humor-, vitreous humor-, and serum concentration-time data were each fit by standard non-compartmental analysis to determine the half-life (*t*_*1/2*_), AUC_0-∞_, and conbercept vitreous clearance using WinNolin Pro (version 8.3.1, Pharsight, Mountain View, CA). Initial parameter estimates were calculated by nonlinear least squares regression analysis using WinNolin Pro version 8.3.1.

## Results

### Pharmacokinetics of Conbercept in the Injected Eye

**FIGURE 1:**
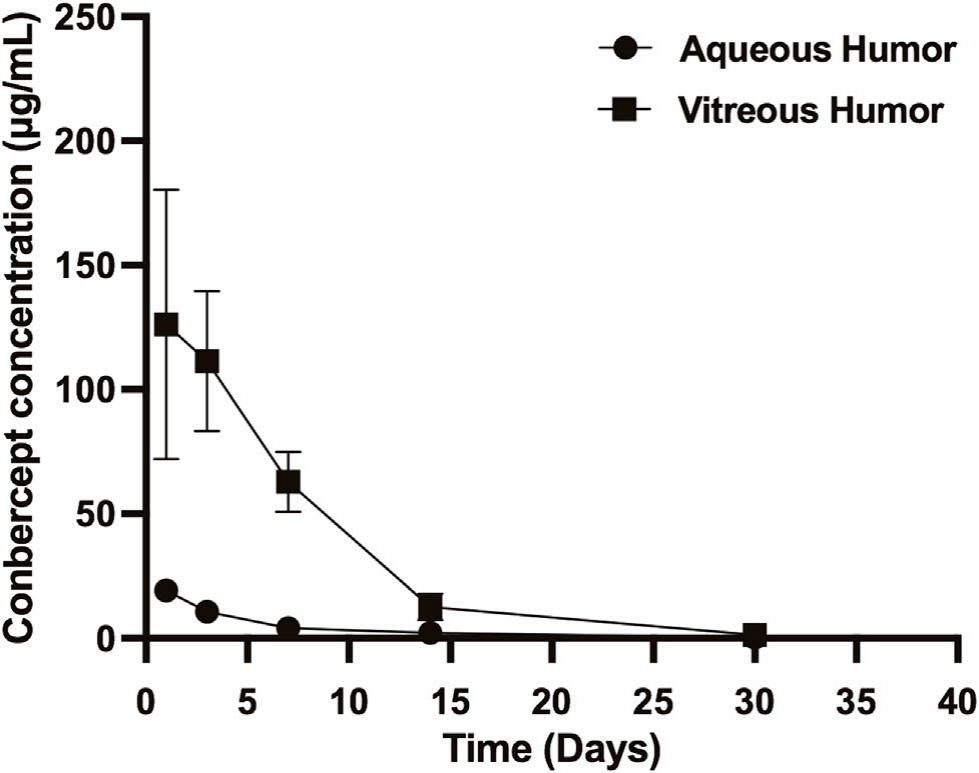
Conbercept concentration in the vitreous humor and aqueous humor after intravitreal injection of 0.5 mg of conbercept. Samples were taken from the aqueous and vitreous of the injected eye.

After 1, 3, 7, 14, and 30 days of intravitreal injection of conbercept (0.5 mg/0.05 ml), the vitreous concentrations of conbercept were 126.25 μg/ml, 111.43 μg/ml, 62.83 μg/ml, 12.69 μg/ml, and 1.63 μg/ml, respectively. Additionally, in the aqueous humor, the conbercept concentrations were 19.21 μg/ml, 10.70 μg/ml, 1.40 μg/ml, 2.03 μg/ml, and 0.10 μg/ml, respectively (Figure 1). One day following intravitreal injection of conbercept, the vitreous humor reached a peak concentration of 126.25 μg/ml. The vitreous concentration of conbercept declined in a monoexponential fashion with a half-life of 4.24 days. Conbercept at a concentration >1 μg/ml was maintained in the vitreous humor for 30 days. The conbercept concentration in the aqueous humor of the injected eye peaked at 19.21 μg/ml 1 day following drug delivery. Conbercept elimination from the aqueous humor had a half-life of 5.68 days, similar to that of the vitreous humor. The maximum concentration of conbercept in the aqueous humor was 15.2% that of the maximum concentration in the vitreous humor, and the total exposure of the aqueous humor to conbercept was 10.79% that of the vitreous humor (Table 1).

**TABLE 1:** Concentration of conbercept in the vitreous, aqueous, and serum after intravitreal injection of 0.5 mg conbercept.

Compartment	t_1/2_ (days)	T_max_ (days)	C_max_ (μg/ml)	% of vitreous C_max_	AUC_inf_ (μg/ml*day)	Exposure to conbercept as a % of vitreous exposure
Vitreous	4.24	1	126.25	_	595,717	_
Aqueous	5.68	1	19.21	15.2	64,251	10.79
Serum	8.95	1	0.100	0.08	512	0.08

t_1/2_ = half-life; T_max_ = time to attain maximum concentration; C_max_ = maximum concentration; AUC = area under curve.

Samples were taken from the aqueous and vitreous of the injected eye.

### Pharmacokinetics of Conbercept in the Non-injected Eye

**FIGURE 2:**
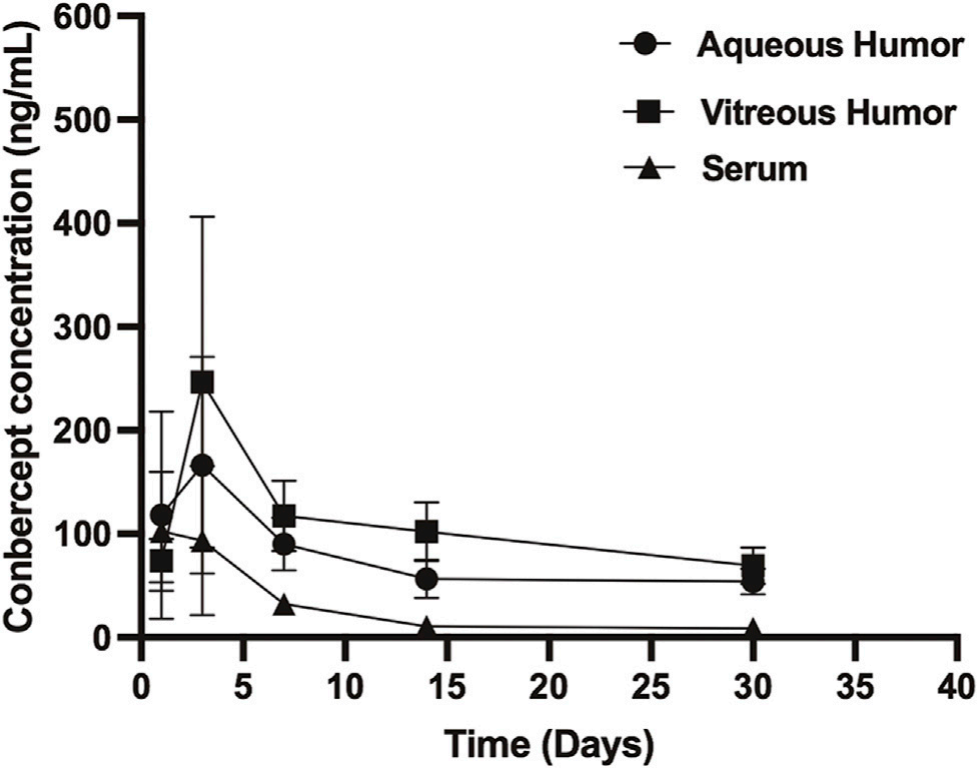
Conbercept concentration in the vitreous humor, aqueous humor, and serum after intravitreal injection of 0.5 mg of conbercept. Samples were taken from the aqueous and vitreous of the non-injected eye.

Conbercept was also detected in the corresponding non-injected eye. The conbercept concentration in the vitreous humor of the non-injected eye increased from 74.11 ng/ml on day 1 to 246.69 ng/ml after 3 days and then decreased to 69.11 ng/ml after 30 days. The level of conbercept in the aqueous humor of the non-injected eye peaked at day 1 with a concentration of 244.82 ng/ml and declined to 40.13 ng/ml after 30 days (Figure 2). The maximum concentrations of conbercept in the vitreous humor and aqueous humor of the non-injected eye were 0.2 and 1.3% those of the injected eye, respectively.

### Pharmacokinetics of Conbercept in Venous Serum

After 1, 3, 7, 14, and 30 days of intravitreal conbercept injection (0.5 mg/0.05 ml), the venous serum concentrations of conbercept were 102.49 ng/ml, 93.61 ng/ml, 32.43 ng/ml, 10.55 ng/ml, and 8.95 ng/ml, respectively. A peak concentration of 102.49 ng/ml was achieved in the venous serum 1 day after intravitreal injection of conbercept, which was 0.08 and 0.5% that of the maximum concentration of conbercept in the vitreous humor and aqueous humor of the injected eye, respectively and 41.5 and 41.8% that of the maximum concentration of conbercept in the vitreous humor and aqueous humor of the non-injected eye, respectively. The venous serum concentration of conbercept declined with a half-life of 8.95 days, and the total exposure of venous serum to conbercept was 0.08% that of the injected eye vitreous humor (Table 1).

## Discussion

Conbercept is a recombinant fusion protein that combines the Fc region of human IgG1 and several ligand-binding domains of human VEGF receptors 1 (Flt-1) and 2 (KDR). In 2008, Zhang et al. (2009) administered a single intravitreal injection of conbercept (0.5 mg) into each eye of six rhesus monkeys to analyze the distribution of conbercept in the eyes. At 2, 7, and 15 days following dosing, two monkeys were sacrificed, and four eyes were enucleated. Concentrations of conbercept in the vitreous humor, aqueous humor, iris, neural retina, and choroid tissues were measured. According to the findings, the vitreous humor had the highest conbercept level, and the surrounding tissue, including the retina and choroid, also had detectable amounts of conbercept 15 days after dosing. However, because the study detected only the conbercept concentration at three time points after intravitreal injection, it was impossible to obtain the intraocular pharmacokinetic profile of conbercept in rhesus monkey eyes. In 2012, Li et al. (2012) investigated the ocular pharmacokinetics of rabbits, following a single intravitreal injection of conbercept (0.5 mg). The study detected the conbercept concentration in the vitreous humor, aqueous humor, and serum at several different time points. The results showed that the peak concentration was 249.22 μg/ml 0.5 days after intravitreal injection with a half-life of 4.24 days. In our study, we obtained the same half-life of conbercept in the vitreous, but the peak concentration of 126.25 μg/ml was achieved in the vitreous humor one day after intravitreal injection of conbercept. This difference might be related to the initial detection time of conbercept concentration that was observed on day 1. Additionally, the half-life of conbercept in the aqueous humor and serum was higher than that found by Li et al. (Li et al., 2012), which might be related to the different breeds of experimental rabbits.

The animals in the studies by Zhang et al*.* (2009) and Li et al*.* (2012) received a single bilateral intravitreal injection of conbercept (0.5 mg/0.05 ml). Thus, the conbercept concentration in the non-injected eyes was not further investigated. Avery et al. (2014) reported the systematic pharmacokinetics of intravitreal injection of ranibizumab, aflibercept, and bevacizumab in 56 patients with neovascular age-related macular degeneration. It was found that aflibercept and bevacizumab could reduce free VEGF levels in serum, and this result was consistent with that of Bakri et al. (2007a, b). Our previous study also found a significant decrease in CRT, and the increased/unchanged BCVA accounted for a high proportion of untreated eyes (67%) after unilateral intravitreal injections of conbercept in bilateral (Di et al., 2020). In the present study, the results showed that conbercept could also be detected in the corresponding non-injected rabbit eyes and serum. The maximum conbercept concentration in the vitreous humor was similar to that in the aqueous humor. Although conbercept has a similar molecular weight (149 kD) to bevacizumab (143 kD), the conbercept concentration in serum was lower than that of bevacizumab, as found in the study by Bakri et al (2007b). This difference might be related to the different single intravitreal injection drug doses. Bakri et al*.* (2007b) gave a single intravitreal injection of 1.25 mg of bevacizumab in unilateral rabbit eyes, which was 2.5 times greater than that of the single intravitreal injection of conbercept (0.5 mg) in our study.

Different kinds of molecules might be related to the low concentration of conbercept in the contralateral eyes. Soler et al. (2017) performed a preliminary study that evaluated the safety and efficacy of medium-chain triglycerides for use as a tamponading agent in minipigs. He found that electroretinography and histological analyses showed no significant difference between medium-chain triglycerides eyes and contralateral eyes. In addition, the molecular weight is a determining factor for ocular pharmacokinetics, according to Dias and Mitra (2000). Because the rate of diffusion is inversely proportional to the cube root of the molecular weight, higher molecular weights are expected to extend the half-life in the vitreous. Moreover, Hanhart et al. (2014) found that the effects of bevacizumab on untreated eyes are related to the Fc portion and its active transport into the systemic circulation. Conbercept includes the Fc region of the immunoglobulin IgG1 with a molecular weight of 149 kD. It was speculated that the molecular type and structure of conbercept were related to its effects on the fellow eye. Khodabande et al. (2021) found some electroretinography changes in contralateral rabbits’ eyes in the unilateral intravitreal injection of digoxin and carboplatin. They proposed it might be related to the repeated intravitreal injection of the agents that cause damage to the ocular-blood barrier. Furthermore, in both eyes of patients who were treated unilaterally for uveitis-related cystoid macular edema, Acharya et al. (2011) found that intravitreal injection of ranibizumab had a beneficial effect. Wu and Sadda (2018) performed unilateral intravitreal injection of bevacizumab and ranibizumab in a patient with bilateral macular edema secondary to branch retinal vein occlusion. After 6 weeks of follow-up, the bilateral macular edema was relieved. Therefore, it has been speculated that the impaired blood-retinal barrier function may alter penetrance to the fellow eye. However, Bakri et al. (2007a, b) discovered that the concentration of bevacizumab in the aqueous humor was higher than that in the vitreous humor in the non-injected eye at time points before 21 days, and he proposed that bevacizumab enters the eye from systemic circulation *via* the anterior route where it diffuses into the vitreous, rather than through the choroidal blood flow. In our study, we found that the maximum conbercept concentrations in the aqueous humor and vitreous humor of non-injected eyes were similar, but the time of peak conbercept concentration in the aqueous humor was earlier than that in the vitreous humor. The conbercept concentration in the serum was lower than that in the aqueous humor and vitreous. These results were consistent with those of Bakri et al. (2007b). In addition, Chou et al. (2018) performed an animal study to confirm previous reports that retrobulbar lidocaine impairs axonal transport; the results showed that fluorescence is absent in the lateral geniculate nucleus (LGN) and superior colliculi (SC) contralateral to the lidocaine-injected eye, but fluorescence is present in the LGN and SC contralateral to the phosphate-buffered saline-injected eyes. Thus, the axonal transport might also be related to the low concentration of conbercept in the contralateral eyes.

However, our study also has limitations. First, there are structural differences between rabbit eyes and human eyes. Rabbits have a less vascular retina, a smaller vitreous cavity (1.5 ml vs. 4.5 ml in humans), a larger lens, and a smaller serum compartment. Because of these factors, pharmacokinetics in rabbits may differ from those in humans. Second, normal healthy rabbit eyes were selected in our study, and an animal model with fundus disease, such as AMD, DME, or macular edema secondary to retinal vein occlusion was not established, which can be further studied in the future. Finally, the initial time for determination of the conbercept concentration for the pharmacokinetic analysis was one day after conbercept intravitreal injection. Since this study mainly observed the distribution of conbercept in the non-injected eyes and previous studies have analyzed the pharmacokinetics of conbercept in the injected eyes, determination of the conbercept concentration was not performed in the early stage after intravitreal injection. However, the ocular pharmacokinetics results from this study are consistent with those of previous studies.

In conclusion, after intravitreal injection of 0.5 mg of conbercept into rabbit eyes, very small amounts of conbercept were detected in the serum and the fellow non-injected eye. In the venous serum, the maximum conbercept concentrations were much lower than those that in the vitreous humor and aqueous humor of the injected eye (0.08 percent and 0.5 percent, respectively). In the non-injected eye, the maximum concentrations of conbercept in the aqueous humor and vitreous humor were similar, at 0.2 and 1.3% of those in the injected eye, respectively.

## Data Availability

The raw data supporting the conclusions of this article will be made available by the authors, without undue reservation.

## References

[B1] Acharya N. R. Sittivarakul W. Qian Y. Hong K. C. Lee S. M. (2011). Bilateral Effect of Unilateral Ranibizumab in Patients with Uveitis-Related Macular Edema. Retina 31, 1871–1876. 10.1097/IAE.0b013e318213da43 21716165

[B2] Avery R. L. Castellarin A. A. Steinle N. C. Dhoot D. S. Joseph Pieramici D. See R. (2014). Systemic Pharmacokinetics Following Intravitreal Injections of Ranibizumab, Bevacizumab or Aflibercept in Patients with Neovascular AMD. Br. J. Ophthalmol. 98, 1636–1641. 10.1136/bjophthalmol-2014-305252 25001321 PMC4251300

[B3] Bakri S. J. Snyder M. R. Reid J. M. Pulido J. S. Ezzat M. K. Singh R. J. (2007b). Pharmacokinetics of Intravitreal Ranibizumab (Lucentis). Ophthalmology 114, 2179–2182. 10.1016/j.ophtha.2007.09.012 18054637

[B4] Bakri S. J. Snyder M. R. Reid J. M. Pulido J. S. Singh R. J. (2007a). Pharmacokinetics of Intravitreal Bevacizumab (Avastin). Ophthalmology 114, 855–859. 10.1016/j.ophtha.2007.01.017 17467524

[B5] Chen X. Li J. Li M. Zeng M. Li T. Xiao W. (2013). KH902 Suppresses High Glucose-Induced Migration and Sprouting of Human Retinal Endothelial Cells by Blocking VEGF and PIGF. Diabetes Obes. Metab. 15, 224–233. 10.1111/dom.12008 22958404

[B6] Chou T. H. Musada G. R. Romano G. L. Bolton E. Porciatti V. (2018). Anesthetic Preconditioning as Endogenous Neuroprotection in Glaucoma. Int. J. Mol. Sci. 19, 237. 10.3390/ijms19010237 PMC5796185 29342845

[B7] Di Y. Li Z. Ye J. Li L. Li B. Yu R. (2020). The Fellow Eye Effect of Unilateral Intravitreal Conbercept Injections in Eyes with Diabetic Macular Edema. Acta Diabetol. 57 (8), 1001–1007. 10.1007/s00592-020-01511-x 32215730

[B8] Dias C. S. Mitra A. K. (2000). Vitreal Elimination Kinetics of Large Molecular Weight FITC-Labeled Dextrans in Albino Rabbits Using a Novel Microsampling Technique. J. Pharm. Sci. 89, 572–578. 10.1002/(SICI)1520-6017(200005)89:5<572:AID-JPS2>3.0.CO;2-P 10756322

[B9] Hanhart J. Tiosano L. Averbukh E. Banin E. Hemo I. Chowers I. (2014). Fellow Eye Effect of Unilateral Intravitreal Bevacizumab Injection in Eyes with Diabetic Macular Edema. Eye (Lond) 28, 646–653. 10.1038/eye.2014.94 24858528 PMC4058632

[B10] Huang C. Y. Lien R. Wang N-K. Chao A-N. Chen K-J. Chen T-L. (2018). Changes in Systemic Vascular Endothelial Growth Factor Levels after Intravitreal Injection of Aflibercept in Infants with Retinopathy of Prematurity. Graefes Arch. Clin. Exp. Ophthalmol. 256, 479–487. 10.1007/s00417-017-3878-4 29290015

[B11] Khodabande A. Ghassemi F. Asadi Amoli F. Riazi-Esfahani H. Mahmoudzadeh R. Mehrpour M. (2021). Ocular Safety of Repeated Intravitreal Injections of Carboplatin and Digoxin: A Preclinical Study on the Healthy Rabbits. Pharmacol. Res. Perspect. 9, e00814. 10.1002/prp2.814 34250764 PMC8273607

[B12] Li H. Lei N. Zhang M. Li Y. Xiao H. Hao X. (2012). Pharmacokinetics of a Long-Lasting Anti-VEGF Fusion Protein in Rabbit. Exp. Eye Res. 97 (1), 154–159. 10.1016/j.exer.2011.09.002 21933673

[B13] Pozarowska D. Pozarowski P. (2016). The Era of Anti-vascular Endothelial Growth Factor (VEGF) Drugs in Ophthalmology, VEGF and Anti-VEGF Therapy. Cent. Eur. Immnol 41 (3), 311–316. 10.5114/ceji.2016.63132 PMC5099389 27833450

[B14] Soler V. J. Laurent C. Sakr F. Regnier A. Tricoire C. Cases O. (2017). Preliminary Study of the Safety and Efficacy of Medium-Chain Triglycerides for Use as an Intraocular Tamponading Agent in Minipigs. Graefes Arch. Clin. Exp. Ophthalmol. 255, 1593–1604. 10.1007/s00417-017-3695-9 28547316

[B15] Wu Z. Sadda S. R. (2018). Effects on the Contralateral Eye after Intravitreal Bevacizumab and Ranibizumab Injections: a Case Report. Ann. Acad. Med. Singap 37, 591–593. 10.1016/j.amepre.2008.04.006 18695773

[B16] Zhang M. Yu D. Yang C. Xia Q. Li W. Liu B. (2009). The Pharmacology Study of a New Recombinant Human VEGF Receptor-Fc Fusion Protein on Experimental Choroidal Neovascularization. Pharm. Res. 26 (1), 204–210. 10.1007/s11095-008-9718-9 18854954

